# Options for Care of Elderly Inpatients With Chronic Diseases: Analysis of Distribution and Factors Influencing Use of Care in Shanghai, China

**DOI:** 10.3389/fpubh.2021.631189

**Published:** 2021-04-29

**Authors:** Jianwei Shi, Ning Chen, Nana Liu, Yan Yang, Dehua Yu, Hua Jin, Li Luo, Xin Gong, Qian Liu, Chen Chen, Wenya Yu, Zhaoxin Wang

**Affiliations:** ^1^Yangpu Hospital, Tongji University School of Medicine, Shanghai, China; ^2^School of Public Health, Shanghai Jiaotong University School of Medicine, Shanghai, China; ^3^School of Medicine, Tongji University, Shanghai, China; ^4^Tongji University School of Economics and Management, Shanghai, China; ^5^Shanghai General Practice and Community Health Development Research Center, Shanghai, China; ^6^School of Public Health, Fudan University, Shanghai, China; ^7^Shanghai Jing'an District Jiangning Road Community Health Service Center, Shanghai, China

**Keywords:** care for elderly people, chronic diseases, elderly inpatients, medical institutions, influencing factors

## Abstract

**Background:** China's ability to provide sufficient healthcare for an elderly population with chronic diseases has become a challenge because of poor utilization of different levels of medical institutions. We aimed to explore the characteristics and factors influencing patient choices and the resulting utilization of different levels of public medical institutions among elderly inpatients with chronic diseases.

**Methods:** Data were collected from the Information Center of the Health and Family Planning Commission of Pudong New Area in Shanghai from 2013 to 2016. A cross-sectional study using multinomial logistic regression analysis was performed to find the factors influencing use of care. Records of patients were identified from electronic health records from public medical institutions.

**Results:** There were 95,445 elderly inpatients with chronic diseases in public medical institutions, 17.78% in community health centers, 68.44% in secondary hospitals, and 13.78% in tertiary hospitals. Compared with those over 80 years old, the 60–69 age group showed a preference for secondary hospitals (OR = 2.980, *P* < 0.001) and tertiary hospitals (OR = 4.497, *P* < 0.001), a trend also observed in the 70–79 age group (OR = 1.353, *P* < 0.001; OR = 1.673, *P* < 0.001). Compared with those using urban employee basic medical insurance, inpatients using urban resident basic medical insurance were less likely to visit secondary hospitals than community health centers (OR = 0.237, *P* < 0.001) or tertiary hospitals (OR = 0.293, *P* < 0.001). Compared with those inpatients who were married, inpatients who were widowed were less likely to go to secondary hospitals (OR = 0.391, *P* < 0.001) or tertiary hospitals (OR = 0.045, *P* < 0.001) than community health centers.

**Conclusions:** The utilization of different levels of medical institutions by elderly people is not well-suited to the respective functions of these medical institutions. Most care services should be provided by community health centers, but our findings indicate that elderly people are more inclined to seek inpatient care at secondary hospitals and tertiary hospitals with some variation based on the patients' sex, age, medical insurance, expenses, and expected length of stay.

## Introduction

In 2016, it was estimated that the number of people aged 60 years and over in China was 230 million, 16.7% of the total population, and that about 150 million had chronic diseases (1). The provision of sufficient healthcare for the population of elderly people with chronic diseases has therefore become a challenge to healthcare in China (2, 3). However, in contrast to some developed countries, there is a shortage of care for the elderly with chronic diseases in different medical institutions in China. In Korea, different settings, such as academic medical centers, community hospitals, ambulatory care clinics, post-acute and long-term care facilities, and palliative care and hospice agencies, can all provide care services for the elderly. Furthermore, doctors in the above institutions can provide either consultation service for clinical medicine, or primary care, or both (4). In Australia, acute services and subacute services (including rehabilitation, geriatric evaluation, and management beds) for the elderly are provided by hospitals, while aged care services are provided by residential aged care facilities, Community Aged Care Packages, Extended Aged Care at Home packages, and the Home and Community Care program. Ensuring that hospitals and aged care institutions are equitably available reduces the possibility of inappropriate care provision in Australia (5). Elderly patients therefore have access to a lot of high-quality medical resources.

Since 2010, China has introduced policies to increase the number of medical institutions providing care for elderly people and improve their service capabilities. The Ministry of Civil Affairs issued a notice on the implementation of the Measures for the Establishment of Permits for Agencies for elderly people and Measures for the Administration of Agencies for elderly people in 2013 (6, 7). This included regulations on the number of beds, the size of funds, the ratio of personnel, and the classification, management, and service content of institutions providing care for elderly people. In 2016, the General Office of the State Council issued a policy to encourage more social and private institutions to provide services specifically for elderly people (8). However, despite these efforts, there are still very few specialized nursing institutions in China. The majority of health services for elderly people like medical care, physical therapy, rehabilitation, and nursing are usually provided by public hospitals and community health centers.

China's policies have specified differentiated roles and functions for each level of the medical institution. The main functions of tertiary hospitals are to provide high-level specialized medical services and treat serious or difficult diseases. Secondary hospitals serve as regional hospitals that provide comprehensive medical and health services for both outpatients and inpatients across many communities. In most cases, tertiary hospitals and secondary hospitals also assume the role of long-term care providers, although with a government-restricted length of stay. Community health centers are primary healthcare institutions that provide basic medical treatment, prevention, rehabilitation, and long-term care directly to the outpatients and inpatients in communities. For inpatient care, the community health centers should not only provide care of common and chronic diseases but also provide hospice care for inpatients (9).

If elderly patients with chronic diseases sought the appropriate levels of medical institution for their needs, the issues of unmatched provision and misutilization of medical services in China could be alleviated. However, little is known about the utilization of different levels of medical institutions by elderly people with chronic diseases. The purpose of our study was therefore to analyze the utilization of different levels of public medical institutions among elderly inpatients with chronic diseases. We also analyzed factors that might affect patients' choices for institutions. We hope the results will improve service utilization for elderly patients with chronic diseases and improve the efficiency of medical institutions in China.

## Methods

### Data Source

Data on elderly patients with chronic diseases were collected from the Information Center of the Health and Family Planning Commission of Pudong New Area in Shanghai. The study included all patients admitted to different levels of public medical institutions in the Pudong New Area, age 60 and older, with chronic diseases from 2013 to 2016. The patient records were identified from the electronic health records of the 57 medical institutions. All the medical institutions from which data were extracted are public medical institutions, and patients had access to all medical institutions. Among all the 57 medical institutions, 40 of them are community health centers, 15 are secondary hospitals, and 2 are tertiary hospitals. We chose samples from this area because Shanghai is economically well-developed in China, and Pudong New Area is typical of Shanghai. Therefore, we used data from Pudong New Area as representative of the chronic disease service capability of public medical institutions in all of Shanghai.

We chose to start with 2013 because it was the 1st year of a sound and unified information system which required all the health institutions to upload data about inpatients. The primary diagnosis in the electronic health record (EHR) was taken as the disease diagnosis for admission to the hospital, and the second to seventh diagnoses were assessed as the basis for the number of complications. In total, the study included 95,445 inpatients with chronic diseases, based on the International Classification of Diseases-9 (ICD-9) and Global Burden of Disease Classification of chronic disease. The sample size (95,445) in this study was the number of patients. For repeat visits in this database, only information related to the first admission was collected. This study was approved by the Ethics Committees of Tongji University (ref: LL-2016-ZRKX-017). Written informed consent was obtained from all participants.

### Analytical Methods

Descriptive statistics to analyze sex, age, medical payment methods, marital status, number of complications, length of stay, total cost, and self-pay in the three levels of public medical institutions (community health centers, secondary hospitals, and tertiary hospitals) were conducted by SPSS 20.0. We used multinomial logistic regression analysis to determine the factors influencing patient choices for medical treatment in the study population.

We set community health centers as a control variable and sex, age, medical payment methods, marital status, number of complications, total cost, self-pay, and length of stay as independent variables. The dependent variable was the level of public medical institution that elderly inpatients with chronic diseases chose to go to. Sex was divided into male or female and age into three groups (60–69 years old, 70–79 years old, and ≥80 years old). The medical payment method was categorized as urban employee basic medical insurance, urban resident basic medical insurance, out-of-pocket, and other payment methods. Other payment methods in this study included the new rural cooperative medical insurance, impoverished rescue, commercial health insurance, free medical care, and other social insurance. Marital status was categorized as married, widowed, or other. Multimorbidity in this study refers to the simultaneous existence of multiple chronic diseases or statuses in a single individual. The number of multimorbidities was divided into none, one, and two or more. Total cost, self-pay, and length of stay were treated as continuous variables because they were factors that could influence which level of medical institution patients sought care at.

## Results

### Demographic Information for Inpatients in Different Levels of Institutions

There were 95,445 inpatients over 60 years of age who were admitted to different levels of public medical institutions in Pudong New Area between 2013 and 2016, with 16,969 (17.78%) in community health centers, 65,321 (68.44%) in secondary hospitals, and 13,155 (13.78%) in tertiary hospitals. Table 1 shows the demographic information for the inpatients in these three levels of institutions. In community health centers, 7,395 (43.58%) inpatients were men and 9,574 (56.42%) were women. In secondary hospitals, 32,610 (49.92%) inpatients were men and 32,711 (50.08%) were women. In tertiary hospitals, 6,735 (51.20%) inpatients were men and 6,420 (48.80%) were women. The largest age group of inpatients in community health centers (10,067, 59.33%) and secondary hospitals (26,278, 40.23%) was patients over 80 years of age, while the largest group in tertiary hospitals was patients aged 60–69 years (6,094, 46.32%).

**Table 1:** Demographic characteristics of elderly inpatients with chronic diseases in different institutions during 2013–2016 (*n* = 95,445).

**Variable**	**Classification**	**Community health centers (*****N*** **=** **16,969, 17.78%)**		**Secondary hospitals (*****N*** **=** **65,321, 68.44%)**		**Tertiary hospitals (*****N*** **=** **13,155, 13.78%)**	
		***n***	**%**	***n***	**%**	***n***	**%**
**Gender**							
Male	7,395	43.58	32,610	49.92	6,735	51.20
Female	9,574	56.42	32,711	50.08	6,420	48.80
Sig	<0.001
**Age (years)**							
60–69	2,292	13.51	19,232	29.44	6,094	46.32
70–79	4,610	27.17	19,811	30.33	4,011	30.49
≥80	10,067	59.33	26,278	40.23	3,050	23.19
Sig	<0.001
**Medical payment method**							
Urban employee basic medical insurance	5,758	33.93	46,796	71.64	8,002	60.83
Urban resident basic medical insurance	3,890	22.92	8,912	13.64	1,596	12.13
Out-of-pocket	2,026	11.94	4,978	7.62	2,359	17.93
Other payment methods	5,295	31.20	4,635	7.10	1,198	9.11
Sig	<0.001
**Marital status**							
Married	12,959	76.37	59,798	91.54	13,043	99.15
Widowed	3,339	19.68	3,982	6.10	26	0.20
Other	671	3.95	1,541	2.36	86	0.65
Sig	<0.001
**Number of multimorbidities**							
0	2,303	13.57	6,024	9.22	2,346	17.83
1	2,797	16.48	9,973	15.27	2,722	20.69
≥2	11,869	69.95	49,324	75.51	8,087	61.47
Sig	<0.001
Length of stay^†^ (days)	55.89		24.29		9.83	
Sig	<0.001
Total cost (RMB)^†^	6,965.17		14,865.65		18,585.26	
Sig	<0.001
Self-pay (RMB)^†^	86.61		904.99		770.78	
Sig	1.000

† *Mean*.

In all levels of institutions, the majority of patients were using the urban employee basic medical insurance (community health centers, 33.93%; secondary hospitals, 71.64%; tertiary hospitals, 60.83%). More patients paid out-of-pocket in tertiary hospitals (17.93%) than in community health centers (11.94%) and secondary hospitals (7.62%). Most patients were married (tertiary hospitals, 99.15%; secondary hospitals, 91.54%; community health centers, 76.37%), and those who were widowed tended to choose secondary hospitals over other levels of institutions. Patients with two or more chronic disease complications were more likely to go to secondary hospitals (*n* = 49,324) than other institutions (community health centers, *n* = 11,869; tertiary hospitals, *n* = 8,087).

There were clear differences in average length of stay in three levels of institutions (55.89 days in community health centers, 24.29 days in secondary hospitals, 9.83 days in tertiary hospitals). Tertiary hospitals had an average cost of hospitalization of 18,585 RMB and an average self-pay cost of 770 RMB. Secondary hospitals had an average cost of hospitalization of 14,865 RMB and an average self-pay cost of 904 RMB. Community health centers had an average cost of hospitalization of 6,965 RMB and an average self-pay cost of 86 RMB.

### Multinomial Logistic Regression Analysis of Elderly Inpatients' Use of Different Levels of Public Medical Institutions

Table 2 shows that men were more likely to choose community health centers than secondary hospitals (OR = 0.873, *P* < 0.001) or tertiary hospitals (OR = 0.840, *P* < 0.001). Compared with those over 80 years old, the 60–69 age group and the 70–79 age group were more likely to go to secondary hospitals (60–69 age group: OR = 2.980, *P* < 0.001; 70–79 age group: OR = 1.353 *P* < 0.001) and tertiary hospitals (60–69 age group: OR = 4.497, *P* < 0.001; 70–79 age group: OR = 1.673, *P* < 0.001) than community health centers.

**Table 2:** Multinomial logistic regression analysis of aged inpatients' choice during 2013–2016 (*n* = 95,445).

**Variable**	**Classification**	**Secondary hospitals (Ref.=** **community health centers)**			**Tertiary hospitals (Ref.=** **community health centers)**		
		**β**	***P***	**OR**	**β**	***P***	**OR**
**Gender (Ref.=** **Female)**							
Male		−0.136	<0.001	0.873	−0.174	<0.001	0.840
**Age (Ref.≥80)**							
60–69		1.092	<0.001	2.980	1.503	<0.001	4.497
70–79		0.303	<0.001	1.353	0.515	<0.001	1.673
**Medical payment method (Ref.=** **urban employee basic medical insurance)**							
Urban resident basic medical insurance		−1.440	<0.001	0.237	−1.229	<0.001	0.293
Out-of-pocket		−0.596	<0.001	0.551	−0.026	0.595	0.975
Other payment methods		−2.254	<0.001	0.105	−1.830	<0.001	0.160
**Marital status (Ref.=** **married)**							
Widowed		−0.939	<0.001	0.391	−3.112	<0.001	0.045
Other		−0.468	<0.001	0.626	−1.367	<0.001	0.255
**Number of multimorbidities (Ref.≥2)**							
0		−0.189	<0.001	0.828	0.284	<0.001	1.329
1		0.077	0.031	1.081	0.326	<0.001	1.386
Length of stay (days)		−0.070	<0.001	0.933	−0.173	<0.001	0.841
Total cost (RMB)		0.0001	<0.001	1.000	0.0001	<0.001	1.000
Self-pay (RMB)		0.002	<0.001	1.002	0.002	<0.001	1.002

Table 2 also shows that compared with those using urban employee basic medical insurance, inpatients using urban resident basic medical insurance were more likely to choose community health centers than secondary hospitals (OR = 0.237, *P* < 0.001), as well as tertiary hospitals (OR = 0.293, *P* < 0.001). Inpatients who paid medical expenses themselves (out-of-pocket) tended to choose community health centers over secondary hospitals (OR = 0.551, *P* < 0.001). For those who paid medical expenses with other payment methods, they were more inclined to choose community health centers than secondary hospitals (OR = 0.105, *P* < 0.001) or tertiary hospitals (OR = 0.160, *P* < 0.001). Compared with those inpatients who were married, inpatients who were widowed were more likely to go to community health centers (secondary hospital: OR = 0.391, *P* < 0.001; tertiary hospitals: OR = 0.045, *P* < 0.001).

Between community health centers and secondary hospitals, inpatients without chronic complications were more likely to go to community health centers (OR = 0.828, *P* < 0.001) when compared to inpatients who had two or more chronic disease complications. However, between community health centers and tertiary hospitals, inpatients without chronic complication were more likely to go to tertiary hospitals (OR = 1.329, *P* < 0.001). Inpatients with one chronic complication were more likely to go to tertiary hospitals as well (OR = 1.386, *P* < 0.001).

Inpatients in community health centers had a longer length of stay than those in secondary hospitals (OR = 0.933, *P* < 0.001) and tertiary hospitals (OR = 0.841, *P* < 0.001). The cost of self-pay was higher in secondary hospitals (OR = 1.002, *P* < 0.001) and tertiary hospitals (OR = 1.002, *P* < 0.001).

## Discussion

Few studies have examined the utilization of different levels of Chinese public medical institutions among elderly inpatients with chronic diseases. We found that men were more likely to choose community health institutions. Men showed a preference for community health institutions which may be because males were less likely to practice self-care activities (10) and community health institutions usually provide care for longer periods (11). In addition, males and females may also require different types of care, which might lead to different choices between different genders. Comparing secondary hospitals and community health centers, inpatients with two or more medical complications were more likely to go to secondary hospitals. Between tertiary hospitals and community health centers, inpatients with two or more complications tended to go to community health centers. This preference for community health centers may be due to the inadequate length of stay the tertiary hospitals can provide. Inpatients' self-pay contribution was higher in secondary hospitals and tertiary hospitals than in community health centers, possibly correlated with the higher surgery cost in secondary hospitals and tertiary hospitals.

Compared with those over 80 years old, the 60–69 age group and the 70–79 age group were more inclined to go to secondary hospitals and tertiary hospitals. The reason for this may be related to the limited length of stay permitted by insurance in secondary hospitals and tertiary hospitals. Healthcare for elderly people over 80 years old likely required longer periods for the treatment of chronic and acute diseases, rehabilitation, and follow-up care, regardless of where they were admitted. When discharged from secondary hospitals or tertiary hospitals, patients over 80 likely needed continued post-discharge medical care in community health centers due to either poor health status or the lack of a caregiver in their home. Furthermore, community health centers are located in the community, making them closer and more convenient for elderly patients to access (12).

Hansen et al. (13) and Saeed et al. (14) reported that health insurance, health status, income, and other demographic factors were strong determinants of healthcare utilization (13, 14). These previous studies support our finding that the medical insurance payment method influences how elderly patients choose medical institutions to some extent. Compared with those using urban employee basic medical insurance, elderly inpatients who used urban resident basic medical insurance were more likely to go to community health centers than secondary hospitals and tertiary hospitals. Also, more elderly inpatients who paid medical expenses out-of-pocket chose secondary hospitals than community health centers. Elderly inpatients using other payment methods also showed a preference for community health centers. These results indicate that medical payment methods affect elderly inpatient choices of medical institutions. Medical payment reform is needed to better inform where patients seek healthcare.

Although the roles and functions of different levels of medical institutions have been officially delegated by Chinese policies, there has been little enforcement. This permits the elderly to select their preferred level of medical institution based on insurance, convenience, or predicted length of stay while disregarding an institution's ability to treat their actual medical needs. One study found that elderly people often occupied beds for a long time but only used common healthcare services (15), such as cleaning and hygiene services, diet nursing, care for patients with urination and defecation disorders, and so on (16). Elderly people occupying beds for a long time but only using common healthcare services had lower daily medical expenses but contributed to inpatient bed shortages in public medical institutions with geriatric care. This inefficient utilization of medical resources by the elderly reduced the income for institutional inpatient services, severely inhibiting the growth of institutional business income (17). There is an urgent need for different levels of medical institutions to have specialized roles and functions for the elderly. As a result, elderly patients can better recognize and select the institutions that are suitable for their medical needs.

The construction of an improved care model for elderly patients with chronic diseases is still in its infancy in China. For instance, community health centers in China are assigned to undertake the significant responsibility of basic public health services, health education and promotion, health management for the elderly, health management for patients with chronic diseases, and so on (18). However, the actual utilization of different levels of medical institutions by elderly people does not correspond with the roles and functions of each institution. A study conducted in China reported that elderly patients preferred services provided by secondary and tertiary hospitals for new diagnoses and follow-up visits (19). A second study, based on the data from the China Health and Retirement Longitudinal Study (CHARLES), found that among 10,172 elderly patients, 57.63% of the outpatients chose the community health centers for medical treatment, while only 17% of inpatients chose community health centers for medical treatment. Although community health centers provided convenient services for the elderly, patients were still inclined to choose secondary and tertiary hospitals as their preferred option for inpatient services (20). Wang (21) indicated that the urban employee basic medical insurance and the urban resident basic medical insurance could guide the elderly to choose different medical institutions for medical treatment to some extent. These findings suggest that, along with the clear roles for different levels of medical institutions, medical insurance should also be further refined in order to better guide the elderly in selecting suitable institutions for their specific medical needs.

This study had some limitations. The EHR data source does not include factors such as inpatient income and living environment, both of which may impact the choice and use of resources by inpatients in care institutions. The sample of the study was collected from one city in China, and therefore may not represent the choices of elderly inpatients with chronic diseases in all of China. However, Shanghai has a high proportion of elderly residents and so our findings may be of use to other similar areas or countries. In the future, it will be necessary to repeat this study across China to consider regional differences. Currently, the low number of private healthcare institutions limits our investigation to public medical facilities; however, it is our interest to compare these two groups in future studies.

## Conclusion

The utilization of different levels of public medical institutions by elderly inpatients with chronic diseases is imbalanced. Most of the care services should be provided by community health centers, but data from public medical institutions indicate that elderly patients are more inclined to visit secondary hospitals and tertiary hospitals. While the patient's age and sex as well as policy factors such as medical insurance, expenses, and expected length of stay affect this trend, the overall utilization of various medical institutions by elderly people is not well-suited with the respective functions of each institution. The Chinese government should therefore strengthen medical insurance for community health centers, improve the capacity of community health centers, and ensure that both medical facilities and elderly people with chronic diseases recognize the distinct care functions of each level of the public medical institution.

## Data Availability Statement

The data analyzed in this study is subject to the following licenses/restrictions: The datasets used and/or analyzed during the current study are available from the corresponding author on reasonable request. Requests to access these datasets should be directed to supercell002@sina.com.

## Ethics Statement

The studies involving human participants were reviewed and approved by Ethics Committees of Tongji University (ref: LL-2016-ZRKX-017). The patients/participants provided their written informed consent to participate in this study.

## Author Contributions

JS and NC conceived, coordinated the study, and refined the manuscript. ZW, WY, and CC gave final approval of the version to be published, provided constructive comments, and is the corresponding author. NL, DY, HJ, and LL collected data. NL, YY, XG, and QL interpreted the data. NC performed the statistical analysis and drafted the manuscript. All authors read and approved the final manuscript.

## Conflict of Interest

The authors declare that the research was conducted in the absence of any commercial or financial relationships that could be construed as a potential conflict of interest.
